# The content of delusions in a sample of South African Xhosa people with schizophrenia

**DOI:** 10.1186/s12888-017-1196-3

**Published:** 2017-01-24

**Authors:** Megan M. Campbell, Goodman Sibeko, Sumaya Mall, Adam Baldinger, Mohamed Nagdee, Ezra Susser, Dan J. Stein

**Affiliations:** 1 Department of Psychiatry and Mental Health, University of Cape Town, J-Block, Groote Schuur Hospital, Observatory, Cape Town, 8000 South Africa; 2 Department of Psychology, Rhodes University, Grahamstown, 6140 South Africa; 3 Mailman School of Public Health, Columbia University and New York State Psychiatric Institute, New York, USA; 4 Department of Psychiatry and Mental Health, MRC Unit on Risk and Resilience in Mental Disorders, University of Cape Town, J-Block, Groote Schuur Hospital, Observatory, Cape Town, 8000 South Africa

**Keywords:** Schizophrenia, Delusions, Illness explanations, South Africa, Xhosa people

## Abstract

**Background:**

Although the relationship between cultural beliefs and schizophrenia has received some attention, relatively little work has emerged from African contexts. In this study we draw from a sample of South African Xhosa people with schizophrenia, exploring their cultural beliefs and explanations of illness. The purpose of the article is to examine the relationship between this cultural context and the content of delusions.

**Methods:**

A sample comprising 200 Xhosa people with schizophrenia participating in a South African schizophrenia genomics study were interviewed using the Structured Clinical Interview for DSM-IV Axis I Disorders (SCID-I). Participant delusions were thematically analyzed for recurring themes.

**Results:**

The majority of participants (*n* = 125 72.5%) believed that others had bewitched them in order to bring about their mental illness, because they were in some way jealous of the participant. This explanation aligns well with the understanding of jealousy-induced witchcraft in Southern African communities and highlights the important role that culture plays in their content of delusions.

**Conclusions:**

Improved knowledge of these explanatory frameworks highlights the potential value of culturally sensitive assessment tools and stigma interventions in patient recovery. Furthermore such qualitative analyses contribute towards discussion about aspects of delusional thought that may be more universally stable, and those that may be more culturally variable.

## Background

The core symptoms of schizophrenia have shown consistency across cultural contexts [1, 2], and while the categories of types of delusions (e.g.,: persecutory, grandiose etc.) appear stable, they demonstrate cultural plasticity in their content [3, 4]. For example, transcultural studies have identified universal delusional experiences amongst people with schizophrenia [5] with persecutory delusions being the most commonly reported across a diverse range of samples drawn from Austria [6]; China, Japan and South Korea [7]; India [8]; Lithuania [9]; Pakistan [10] Turkey [11] and the United States [12]. However socio-cultural factors such as religious belief systems [6, 13, 14] and more collective or individualistic conceptualizations of self [15] as well as environmental factors [16], historical events [12] and political contexts [7,9,] have been shown to influence the content of delusions. Within particular cultural groups factors such as sex, age, education and socio-economic status [8, 10], geography [11, 15], urban and rural living contexts [6, 8] have also demonstrated influence. Consistent with international cross-cultural studies, persecutory delusions, including the belief that others are plotting against or planning to hurt the individual, appear to be the most frequently reported type of delusion in South African Xhosa people with schizophrenia [17, 18]. However, to date qualitative exploration of the content of these delusions remains relatively limited. It’s likely that the widely accepted belief in the Xhosa culture that mental illness has its root cause in witchcraft plays an integral role in the content of these delusions [18, 19]. These cultural beliefs also have a broader impact on how schizophrenia is explained and understood in the Xhosa community, influencing help-seeking behaviors and treatment, as well as medication compliance [17, 20, 21].

Improved understanding of the content of persecutory and other delusions of Xhosa people with schizophrenia has implications for better empathy and understanding of these patients and potentially their treatment needs. Furthermore, little is currently known about the interaction between culture and the formation of delusions [3]. Some aspects of delusional thought may be the result of cognitive processes that are biological and universal human experiences, others may demonstrate considerable cultural variability [3, 4]. Consideration of aspects of delusional thought across different cultural contents may prove valuable to the cognitive theory of delusions. This paper aims to contribute to understanding about the relationship between culture and delusional content by exploring the content of delusions reported within a sample of 200 Xhosa people with schizophrenia recruited for a genomics of schizophrenia study in South Africa.

### The Xhosa community

The Xhosa are South African Bantu-speakers, forming part of the Nguni language tribes, whose ancestors migrated to southern Africa over the last 1500 years, and represent both Bantu-speaking African populations and, through admixture, the ancient Khoisan lineage [22]. The Xhosa people fall historically within the group of racially segregated and oppressed South Africans discriminated against during Apartheid governance. As a result many still contend with extreme inequalities with respect to education and economic opportunities, housing and healthcare services amongst others [23]. Xhosa people with schizophrenia are a small minority within this larger community, and contend with the discrimination and stigmatization associated with mental illness, as an added burden [20, 24, 25].

A diverse range of spiritual beliefs underpin the Xhosa culture including African traditional healing methods and medicine, ancestor worship, magic and witchcraft, along with Christian, Islamic, atheist and agnostic beliefs [17]. Witchcraft is particularly prevalent in congested or over-crowded areas [27] where it is believed to result from the ancestors withdrawing their protection, allowing witches to bring misfortune onto an individual [26, 28]. Accusations of bewitchment typically follow a specific circumstance or misfortune, where witchcraft, performed on the grounds of jealousy, is intended to prevent a person from succeeding in life [26, 27]. Communities use the bewitchment framework to explain why misfortune impacts on a specific individual at that particular time [26].

Bewitchment takes numerous forms. One of these is “isidliso” or poisoning by ingestion which may result in physical symptoms such as chronic coughing, chest pain and blood in the sputum, much like Tuberculosis [26, 29]. Bewitchment also takes the form of possessions by evil spirits, the manifestation of which may be consistent with mental illness. One example is “amafufunyana” which refers to a state of perceived possession by multiple spirits that may speak or communicate through the possessed individual [21]. Another state of bewitchment may involve the "dirty spirit," known as the “Tikoloshe”, described as a small hairy man with a large penis which he carries over his left shoulder [30]. The impact of bewitchment may then manifest in social misfortunes such as unemployment, family conflict and a loss of social support within the community [26].

Traditional healing interventions are sought to repel the bewitchment and protect against further attack [26, 29]. Such interventions include acts aimed at cleansing the patient and their family of evil spirits [20, 31]. Biomedical interventions are considered necessary by some patients, their families and traditional healers, in treating the symptoms of any illness resulting from bewitchment [26, 29]. Psychiatric assessment, hospitalization and medication are therefore accepted by some as effective ways of managing psychotic symptoms thought to be induced by witchcraft [20, 32].

### The Genomics of Schizophrenia in South African Xhosa People (SAX) project

Current genetic research under-represents the African lineage [33], making findings that shape our understanding of the underlying causes, prevention, and treatment of schizophrenia less directly applicable to African populations. The Genomics of Schizophrenia in South African Xhosa People (SAX) project seeks to identify genetic variations or mutations underlying predisposition to schizophrenia in the Xhosa people. The SAX project, initiated in January 2013, aimed to recruit 1100 Xhosa people with a clinical diagnosis of schizophrenia or schizoaffective disorder and 1100 matched controls over a 5-year period. Participants were recruited from provincial hospitals and clinics in the Eastern and Western Cape provinces of South Africa.

## Methods

The aim of this article was to explore the content of delusions reported by a sample of Xhosa people with schizophrenia, recruited for the SAX study. The sample included the first 200 participants who were recruited between January and July 2015 on the SAX study across both the Eastern and Western Cape provinces of South Africa. The Eastern Cape is typically associated with rural, traditional Xhosa contexts, while the Western Cape is considered more urban. However, the population tends to migrate between the Eastern Cape where family relatives are based, and the Western Cape where most work opportunities are.

### Recruitment and data collection

A team of five Xhosa psychiatric nurses managed the recruitment process, supervised by a medical doctor working in psychiatry, proficient in Xhosa. During recruitment, participants received information about the SAX study aims, methods and expected outcomes, explained to them in Xhosa. Level of understanding of elements of the study and capacity to consent to participate were evaluated using the University of California, San Diego Brief Assessment of Capacity to Consent Questionnaire (UBACC) [34]. Participants then completed a clinical assessment in Xhosa comprising the Structured Clinical Interview for DSM-IV Axis I Disorders (SCID-I) [35], along with a neurocognitive battery [36] and other measures such as socio-demographic instruments, the Childhood Trauma Questionnaire [37] and the Discrimination and Stigma Experiences Scale [38]. Participants also provided blood samples for DNA and HIV testing. All clinical interview materials, neurocognitive measures and psychosocial scales were translated into Xhosa in accordance with the World Health Organization (WHO) translation guidelines [39].

Participants were asked to describe their beliefs in response to the SCID-I Module B questions [37]. The SCID-I is a semi-structured interview used for making the DSM-IV Axis I diagnoses. Module B is used to document psychotic and associated symptoms over the person’s lifetime. Delusions are categorized according to their form or type [35] and include a series of questions relating to each category. Explanations of these categories and related questions asked to participants to identify their delusions are outlined in Table 1. During recruitment, delusions and hallucinations were documented in detail in Module B. It is not always easy to distinguish between various types of delusions, and assigning a patient’s delusional beliefs to one or another category is a clinical judgement. As a result, all participant files were reviewed by the second author (GS), the supervising medical doctor with psychiatric training, to ensure that a) participants had been correctly diagnosed during recruitment, b) delusions and hallucinations had been accurately differentiated and that c) delusion type had been consistently categorized. Participant descriptions were translated back into English and recorded by the psychiatric nurses administering the SCID-I.

**Table 1:** SCID-I Module B Questions about delusions

Delusions of reference: belief that the individual is attaching personal inference to events in their environment, e.g., believing that others are talking about or taking special notice of them, or that the individual is receiving special messages from random events around them.	
1a.	Has it ever seemed like people were talking about you or taking special notice of you – describe
1b.	What about receiving special messages from the TV, radio, or newspaper, or from the way things were arranged around you - describe
Persecutory delusions: belief that others are plotting to actively harm or disadvantage them	
2.	What about anyone going out of their way to give you a hard time, or trying to hurt you – describe
Grandiose delusions: beliefs of possessing great wealth, importance or special powers	
3.	Have you ever felt that you were especially important in some way or that you had special powers to do things that other people could not do – describe
Somatic delusions: beliefs of abnormal functioning or anatomy of the individual’s body and body parts despite medical evidence to the contrary	
4a.	Have you ever felt that something was very wrong with you physically even though your doctor said nothing was wrong – describe
4b.	Have you ever been convinced that something was very wrong with the way a part or parts of your body looked – describe
4c.	Have you ever felt that something strange was happening to parts of your body – describe
Other delusions: unusual religious experiences, unexplained excessive guilt, jealousy or the false perception that someone famous or well known is in love with the individual	
5a.	Have you ever had any unusual religious experiences – describe
5b.	Have you ever felt that you had committed a crime or done something terrible for which you should be punished- describe
5c.	Were you ever convinced that your spouse or partner was being unfaithful to you - describe
5d.	Did you ever feel you had a special, secret relationship with someone famous or someone you didn’t know very well - describe
Delusions of being controlled: belief that an external force or person is controlling their thoughts and actions	
6a.	Did you ever feel that someone or something outside yourself was controlling your thoughts or actions against your will – describe
6b.	Did you ever feel that certain thoughts that were not your own were put into your head or taken out of your head – describe
Thought broadcasting: belief that their thoughts are perceptible to those around them	
7a.	Did you ever feel as if your thoughts were being broadcast out loud so that other people could actually hear what you were thinking – describe?
7b.	Did you ever believe that someone could read your mind – describe?

### Data analysis

These responses were then extracted from the SCID-I, tabulated and analyzed for recurring themes by the first author (MC). Categories were used to identify: i) the delusions most commonly reported within this sample and ii) specific themes within the content of these delusions. The resultant categories and their content were reviewed by the second (GS) and third (SM) authors to ensure consistency in interpreting the emerging themes presented in Table 3.

## Results

The sample of 200 Xhosa people with schizophrenia recruited for the SAX study included 104 (52%) in-patients and 96 (48%) out-patients from Eastern (*n* = 142, 71%) and Western (*n* = 58, 29%) Cape provincial psychiatric hospitals and out-patient clinics in South Africa. Of the total sample, 168 (84%) were male and 32 (16%) were female, ranging in age from 20-54 years with a mean age of 35 years. Of the total sample, most participants (187, 93.5%) were non-reactive to HIV testing. Majority of the sample (*n* = 83, 41.5%) reported their highest level of education between Grades 1-7, while 57 (28.5%) had completed Grades 8-10 and 53 (26.5%) Grades 11-12. Only 7 (3.5%) participants had participated in higher education or training.

The total sample included 197 (98.5%) Xhosa people who met the diagnostic criteria for schizophrenia, of which over half were paranoid type (*n* = 105, 52.5%). The other 3 (1.5%) participants met the criteria for schizoaffective disorder. The majority of participants (*n* = 128, 64%) were assessed as having moderate symptom severity, while most participants’ (*n* = 121, 60.5%) Global Assessment of Functioning (GAF) scores fell in the 31-70 point range. Over half of the total sample (*n* = 120, 60%) met criteria for a co-morbid substance-use disorder. Of these cannabis (*n* = 55, 46%), alcohol (*n* = 16, 13%), cannabis and alcohol (*n* = 35, 29%) were the most frequently reported substances. These demographics are summarized in Table 2. Resultant themes of the analysis of the content of delusions are presented in Table 3 and described below.

**Table 2:** Sample demographics: Xhosa people with schizophrenia (*n* = 200)

Patient care	In-patients: 104 (52%)	Out-patients: 96 (48%)
Region	Eastern Cape: 142 (71%)	Western Cape: 58 (29%)
Sex	Male: 168 (84%)	Female: 32 (16%)
Age	Range: 20-54	Average age: 35 years
Education	Diploma/Degree: 7 (3.5%)	
	Grades 11-12: 53 (26.5%)	
	Grades 8- 10: 57 (28.5%)	
	Grades 1-7: 83 (41.5%)	
Diagnosis Schizophrenia *n* = 197 (98.5%)	Paranoid *n* = 105 (52.5%)	
	Undifferentiated *n* = 39 (19.5%)	
	Disorganized *n* = 28 (14%)	
	Residual *n* = 22 (11%)	
	Other *n* = 3 (1.5%)	
Schizoaffective *n* = 3 (1.5%)	Bipolar *n* =2 (1%)	
	Depression *n* = 1 (0.5%)	
Symptom severity	Full remission *n* = 23 (11.5%)	
	Mild *n* = 40 (20%)	
	Moderate *n* = 128 (64%)	
	Severe *n* = 2 (1%)	
	Not reported *n* = 7 (3.5%)	
Global Assessment of Functioning (GAF) scores	0-30 *n* = 25 (12.5%)	
	31-70 *n* =121 (60.5%)	
	71-90 *n* = 39 (19.5%)	
	91-100 *n* = 0	
	Not reported *n* = 15 (7.5%)	
Co-morbidity	Substance use *n* = 120 (60%)	
	*Cannabis *n* = 55 (46%) *Alcohol *n* = 16 (13%)	
	*Cannabis and Alcohol *n* = 35 (29%)	
	*Other *n* = 16 (13%)

**Table 3:** Themes of the content of delusions from a sample of 200 Xhosa people with schizophrenia (subgroups are not mutually exclusive)

1. Delusions of reference	2. Persecutory delusions	4. Somatic delusions
People talking about them: *n* = 127 (63.5%) a) Who were these people? (*n* = 127 responses) • People in general *n* = 84 (66%) • Family/friends/neighbors *n* = 44 (36%) • Colleagues/patients/hospital staff *n* = 19 (15%) b) What were they doing? (*n* = 62 responses) • Looking at them strangely *n* = 35 (56%) • Saying bad things/laughing at them *n* = 31 (50%) • Actively plotting against them *n* = 6 (10%) c) How did you react? (*n* = 22 responses) • Passive reaction: withdraw/avoid *n* = 13 (59%) • Aggressive reaction: fight/attack *n* = 8 (36%) • No reaction *n* = 1 (4.5%) Receiving special messages: 105 (52.5%) a) Where were the messages from? (*n* = 105 responses) ▪ TV *n* = 92 (87.5%) ▪ Radio *n* = 32 (30.5%) ▪ Other (e.g.,: magazines/newspapers) *n* = 7 (6.5%) b) What was the message about? (*n* = 105 responses) • Talking to them *n* = 72 (68.5%) • Talking about them *n* = 40 (38%) • Playing out their life story/showing images of them/experiences of being inside the medium *n* = 18 (16.5%) c) How did you react? (*n* = 15 responses) • Passive/withdraw *n* = 2 (13.5%) • Aggressive/attack *n* = 3 (20%) • Assertive/speak back/turn off *n* = 10 (66.5%)	People plotting against you: *n* = 172 (86%) a) In the form of bewitchment *n* = 125 (72.5%) • Using a Tokoloshi *n* = 4 • Using Amafufunyana *n* = 12 b) Who were these people? (*n* = 172 responses) • People in general *n* = 65 (38%) • Family/friends/neighbors *n* = 102 (59%) • Colleagues/patients/hospital staff *n* = 15 (8.5%) c) Why were they doing this? (*n* = 78 responses) • Jealousy *n* = 71 (91%) • People don’t like me *n* = 6 (7.5%) • These people are bewitched *n* = 1 (1.5%) d) Did you seek help from anyone? (*n* = 21 responses) • Sangoma/Traditional healer *n* = 19 (90.5%) • Other: prophet/ancestor *n* = 2 (9.5%)	Something physically wrong: *n* = 73 (36.5%) • Head/brain/skull *n* = 31 (42.5%) • Stomach *n* = 19 (26%) • Whole body *n* = 10 (13.5%) • Heart *n* = 5 (7%) • Other: eyes/ears/hands/legs *n* = 17 (23%)
		5. Other delusions n = 42 (21%)
		• Relationship with famous person: *n* = 11 (26%) • Special relationship with God: *n* = 11 (26%) • Guilt/should be punished: *n* = 11 (26%) • Convinced partner is unfaithful: *n* = 10 (24%)
		6. Delusions of being controlled *n* = 118 (59%)
		a) Who is controlling thoughts? (*n* = 118 responses) • Spirit/amafufunyana/external force *n* = 42 (35.5%) • People in general *n* = 30 (25.5%) • Family/friends/neighbors *n* = 23 (19.5%) • TV/radio *n* = 8 (7%) • Other *n* = 18 (15%)
	3. Grandiose delusions	
	Special powers: n = 118 (59%) • Wealth/success/fame *n* = 56 (47.5%) • God/son of God/prophet/healer *n* = 47 (40%) • Magical powers *n* = 14 (12%) • Education/intellect/knowledge *n* = 13 (12%) • Powerful political leader *n* = 9 (7.5%)	
		7. Thought broadcasting *n* = 117 (58.5%)
		a) Who was broadcasting your thoughts? (*n* = 118 responses) • People in general *n* = 89 (76%) • TV/Radio *n* = 14 (12%) • Family/friends/neighbors *n* = 10 (8.5%) • Doctors/Traditional healers *n* = 2 (1.5%) • Amafufunyana *n* = 2 (1.5%) b) How did you react? (*n* = 17 responses) • Feeling angry/attack *n* = 6 (35.5%) • Feeling isolated/rejected/criticized *n* = 11 (64.5%)

### Questions relating to delusions of reference

Of the total sample two thirds (*n* = 127, 63.5%) reported the belief that others were talking about them, while half (*n* = 105, 52.5%) believed they were receiving special messages from sources such as televisions, radios and print media. Approximately a quarter of the total sample (*n* = 54, 27%) reported experiencing both these delusions.

### People talking about them

Of those who reported the belief that others were talking about them (*n* = 127, 63.5%), the majority (*n* = 84, 66%) reported these to be people unknown to them personally. However, over a third (*n* = 44, 36%) made reference to their immediate social circle (specific family members, friends and/or neighbors). A smaller group (*n* = 19, 15%) referred to colleagues, other patients and/or hospital staff. A 20-year-old male from the Western Cape explained that “he felt his brother and sister were talking about him whenever they spoke together”. A 32-year-old male from the Eastern Cape reported that “whenever he walked past people he thought they were talking about him, especially when they were laughing”. One 33-year old female from the Eastern Cape explained that “she felt as though people at home and school were talking about her constantly even though she hadn’t done anything for them to talk about”.

In addition to believing people were talking about them, almost half (*n* = 62, 48%) of this group reported that others were reacting to them in negative ways, either by looking at them strangely (*n* = 35, 56%), laughing at them and saying hurtful things about them (*n* = 31, 50%), or actively plotting with others to harm them (*n* = 6, 10%). A 26-year-old female from the Western Cape “believed everyone at high school was talking about her. Peers looked at her in strange ways in class. When they passed her by they would laugh at her”.

Of these individuals, a third (*n* = 22, 35,5%) described their reactions to these experiences. Over half (*n* = 13) reported withdrawing, isolating themselves and avoiding others in response. A 47-year-old man from the Western Cape said that “sometimes he didn’t want to see [and visit] with people because they looked at him in a strange way that made him feel uncomfortable and want to leave”. Others (*n* = 8) reported reacting in aggressive ways such as fighting with, shouting at and/or threatening others. Only 1 person responded that the negative perceived reactions of those around her did not affect her.

### Receiving special messages

Of those who reported the experience of receiving special messages from outside sources (*n* = 105, 52.5%), the majority (*n* = 92, 87.5%) reported receiving messages from televisions, while a third (*n* = 32, 30.5%) made reference to their radios. Of these 105 individuals, many (*n* = 72, 68.5%) reported the belief that these sources were talking to them. A 37-year-old male from the Eastern Cape stated that “people on TV would talk to him and sometimes come out of the TV to visit and chat with him. Everyone on the radio knew him and he would receive special messages from them”. One 50-year-old male from the Eastern Cape explained that “people on TV were threatening him and saying they would burn him alive. He would run away and the people on TV would laugh at him”. A 51-year-old female from the Eastern Cape reported that “people on TV were talking to her and telling her that her husband was cheating on her”.

Over a third (*n* = 40, 38%) of the total sample reported the belief that they were being spoken about. A 28-year old male from the Eastern Cape reported that “people on TV looked at him in strange ways and laughed at him. People on Generations [a popular South African TV series] were talking about him”. A 39-year-old man from the Eastern Cape explained that “people on TV were saying that he was stupid, poor and ugly so he stopped watching”. Other experiences included watching one’s life experiences played out on TV (*n* = 8), the feeling of being inside the TV (*n* = 5) and watching oneself on TV (*n* = 5).

Only 14 individuals commented on how they reacted to these experiences. The majority (*n* = 10) responded either by turning off or talking back to the source. One 38-year-old male from the Eastern Cape explained that “people on the TV show Generations would talk to him. His mother would chase him out the room when watching because he would talk back to the characters. His radio would preach about him. He would wake up in the night and shout back at the people on the radio”. Some people reported (*n* = 3) responding aggressively by attacking or breaking the source, while others responded passively (*n* = 2) by running away.

### Persecutory delusions

Of the total sample, most (*n* = 172, 86%) reported the experience of people plotting against them to hurt or harm them. One 29-year-old male from the Eastern Cape explained that “people were conspiring against him. He could not trust anyone. He feared that people wanted to kill him. He did not feel safe at home”. A 31-year-old male from the Eastern Cape reported that “his friends were plotting against him, making evil traps for him to fall into. He felt they no longer liked him and he was unable to trust any of them”.

Of those found to have persecutory delusions, 125 (72.5%) believed that others had bewitched them in order to bring about their mental illness, through the use of “amafufunyana” and/or the “tikoloshe”. A 31-year-old male from the Eastern Cape explained “that his father was the reason for his illness. His father had used an evil snake to bewitch him”. A 33-year-old male from the Western Cape stated that “he was bewitched and possessed by evil spirits. The tikoloshe was following him. The Sangoma [traditional healer] confirmed that he was filled with evil spirits”. One 49-year-old female from the Eastern Cape reported that “she had first become [mentally ill] when she had amafufunyana inserted into her by the mother of her ex-boyfriend because she was pregnant with his child and the family did not want to pay for the child”.

When asked to identify who was responsible for plotting to hurt, harm and/or bewitch them, the majority (*n* = 102, 59%) of the sample identified family members, friends and neighbours. Over a third (*n* = 65, 38%) made reference to people in the general community, and a small group referred to colleagues, other patients and/or hospital staff (*n* = 15, 8.5%). A 27-year-old male from the Western Cape explained that “he was bewitched by his grandmother who had sent an evil spirit into him. He believed his family was plotting against him and no longer trusted them. He no longer visited with them or attended ceremonies [at his home]”.

Only 78 individuals provided explanations for why they thought others would want to plot against them or bewitch them. Of these, the majority (*n* = 71, 91%) believed others were jealous of them. One 23-year-old male from the Western Cape reported that “he was bewitched by women from his community because he was completing [higher education] while their children were not”. A 31-year-old male from the Eastern Cape believed that “he was bewitched with amafufunyana by his aunt and uncle. They were responsible for his illness. They were jealous of him and did not want him to succeed in life”.

While not asked directly, 21 participants from the total sample volunteered that they had engaged with a Sangoma/traditional healer (*n* = 19), or a prophet or made sacrifices to an ancestor (*n* = 2) in reaction to these persecutory beliefs. A 27-year-old male from the Eastern Cape explained that “he believed he was bewitched and went to see a Sangoma [traditional healer]. The Sangoma told him that there was an evil spirit following him since he was in high school, and gave him muti [traditional herbal medicine]”. One 54-year-old male from the Eastern Cape reported that “his aunt had bewitched him and poisoned his food because she wanted to kill him. [He had visited a] Sangoma who had confirmed that his aunt had poisoned him and put intlanga [evil spirits] in his brain”.

### Delusions of being controlled and thought broadcasting

Of the total sample, over half (*n* = 118, 59%) believed their thoughts were being controlled by an external person or force, while a similar proportion (*n* = 117, 58.5%) reported the belief that their thoughts were being broadcast to others. Approximately a third of the total sample (*n* = 74, 37%) reported experiencing both thought control and thought broadcasting.

### Thought control

Of those who reported the experience of thought control (*n* = 118, 59%), a third (*n* = 42, 35.5%) believed evil spirits and unknown external forces were controlling their thoughts. One 29-year-old male from the Eastern Cape explained that “witches controlled his thoughts and used their voices to control him. His thoughts were not his own”. A 30-year-old female from the Eastern Cape believed that “her thoughts had been inserted by the amafufunyana and all her thoughts were theirs. They controlled her”.

### Thought broadcasting

Of those who were assessed as having experienced thought broadcasting (*n* = 117, 58.5%), the majority (*n* = 89, 76%) believed people unknown to them, residing in their broader community were responsible. One 26-year-old male from the Eastern Cape explained that “people were able to see and hear his thoughts. This would make him feel bad”.

Only 17 individuals commented on how they reacted to these beliefs. Two thirds (*n* = 11) reported feeling isolated, rejected and criticized (*n* = 2). A 23-year-old from the Western Cape believed that “others could hear his thoughts. They could recognize that he was thinking nonsense”. One 30-year-old male from the Eastern Cape explained that “people around him could hear his thoughts. They would then make [unkind] comments about what he was thinking”. The other third (*n* = 6) reported feeling angry and in response, attacking those around them.

### Other delusions

Of the total sample, over half (*n* = 118, 59%) made reference to the belief that they possessed special powers, while a third (*n* = 73, 36.5%) believed something was physically wrong with them despite evidence to the contrary.

### Grandiose delusions

Of those who reported the belief of possessing special stature or powers (*n* = 118, 59%), wealth, success and fame were the most commonly reported (*n* = 56, 47.5%). A 20-year-old male from the Western Cape explained that “he was a very important person, more important than anyone else; and had a lot of money, cars and houses”.

The belief of being God, the son of God, a prophet or a healer was also common (*n* = 47, 40%). One 29-year-old male from the Eastern Cape believed that “he could heal people with his hands and prayers. He reported healing a friend who had been stabbed. He prayed over him and the wound closed”. A 38-year-old male from the Eastern Cape believed “he was able to raise the dead and would spend his time [in graveyards] near graves”.

### Somatic delusions

Of those who believed something was physically wrong with them despite evidence to the contrary (*n* = 73, 36.5%), the head, skull and brain were the most frequently reported areas of the body where these health concerns were experienced (*n* = 31, 42.5%). A 33-year-old male from the Eastern Cape believed that “his brain had been replaced with someone else’s”. A 47-year-old male from the Western Cape “believed that when he was in school his brain was stolen”.

Some individuals also made reference to abnormalities in their stomachs (*n* = 19, 26%). Complaints included the belief that something was crawling in the stomach area. One 20-year-old male from the Western Cape stated that “evil spirits had placed a snake inside his stomach. He believed there was something twisting and turning inside him”. A 45-year-old male explained that “he had a snake in his stomach and believed his intestines were made of snakes”. One 51-year-old male from the Western Cape believed “he had amafufunyana and butterflies flying around in his stomach”.

### Religious or jealous delusions or delusions about guilt

Only a quarter (*n* = 42, 21%) of the total sample made reference to other beliefs such as religious or jealous beliefs or beliefs about guilt. Of the delusions with religious content, beliefs about having a special relationship with God, or a supreme being were the most commonly reported. One 41-year-old female from the Eastern Cape explained that “she was the only person who could speak to God and she taught others how to communicate with Him”.

## Discussion

Persecutory delusions were found to be the most commonly reported type of delusion, consistent with the international literature [5]. In addition delusional content drew from cultural explanations of illness and a bewitchment framework, particularly with respect to persecutory delusions, delusions of reference and somatic delusions. For example, of those who reported persecutory delusions, 72.5% (*n* = 125) believed that others had bewitched them to bring about their mental illness, through evil spirits, “amafufunyana” or the “Tikoloshe”. This bewitchment framework has been reported to influence the content of delusions in other African samples including Sesotho people with schizophrenia from the Free State province of South Africa [Mosotho 40], and in neighboring African countries like Namibia [41], suggesting that this explanatory framework may be common in Southern African cultures. This observation aligns well with epidemiological studies that report the frequent use of spiritual causes as explanations for psychiatric illness in Southern African communities [19]. Three important points emerge from this analysis.

First, a focus on the recurring themes in the content of these delusions provides valuable insight into Xhosa patients’ experiences and potentially their treatment needs, allowing for better empathy and understanding. While participants demonstrated insight into being unwell, it was difficult to determine the extent to which they accepted the label of schizophrenia. Continued research into the content of delusions within African samples may add to increased knowledge and sensitivity about how cultural frameworks of understanding and explaining mental illness impact on the psychotic experiences of these patients. Tools such as the DSM 5 cultural formulation [42] are important aides in bridging the gap between Western and African conceptualisations of psychotic illness experiences. Similarly expanding the bio-psycho-social approach towards illness conceptualization, to include a spiritual element, may play a helpful role in patient recovery [43].

Second, people with schizophrenia represent a vulnerable and socially excluded population who are more likely to be negatively impacted on by poverty, human rights violations, stigma and discrimination [44]. The impact of schizophrenia on an individual’s cognitive, emotional and behavioural functioning may lead to behaviour that draws the attention of others in their community. For example, the referential belief that others were talking about them was the second most frequently reported type of delusion in this sample (*n* = 127, 63.5%), with some patients voluntarily reporting a sense of being reacted to in a negative way by those around them. The belief that illness was caused by bewitchment might also contribute a further layer of stigma [20, 24]. This experience of alienation and othering surely must compound referential thinking. Stigma interventions are therefore highlighted as an important part of the recovery process.

Third, these findings contribute to the debate about aspects of delusional thought that may be more universally stable, and those that may be more culturally specific and variable [3]. One cognitive theory of delusions, proposed by Garety and colleagues suggests that probabilistic reasoning becomes compromised in delusional thinking, leading individuals to jump to conclusions without sufficient evidence [45]. While this may be a more stable aspect of delusional thought, the framework drawn from to explain a person’s anomalous experiences appear to be more culturally variable, as in this study where bewitchment appears as a dominant explanatory framework in the Xhosa community, which may be applicable to other African communities.

One limitation of this study is that it does not document the verbatim responses of participants. However, findings provide a snapshot of the typical content of delusions. A second limitation is that this sample is not representative of all Xhosa people with schizophrenia but rather provides insight into the content of delusions of a sample of patients, the majority of which were male (84%) and living in the Eastern Cape (71%).

Finally, this paper did not examine how these belief effects extend to other psychotic symptoms including hallucinations. The relation of cultural explanations to auditory and tactile hallucinations in Southern Africa is an especially complex topic, because some kinds of hallucinations (e.g., voices of ancestors) are associated with being apprenticed to become Traditional Healers and continue among those who finish their training. Therefore we believe that these hallucinations merit separate treatment; they will be addressed in future work.

## Conclusions

Qualitative exploration of the content of delusions within African samples such as the Xhosa people, contributes to better understanding of the cultural frameworks people draw from in explaining their psychotic illness experiences.

Improved knowledge of these explanatory frameworks has implications for better understanding treatment needs; highlight the value of culturally sensitive assessment tools such as the DSM 5 cultural formulation [42]; and stigma interventions in patient recovery. In addition, qualitative analysis of the content of delusions within specific cultural groups contributes to discussion about aspects of delusional thought that may be more universally stable, and those that may be more culturally variable.

## Abbreviations

SCID-I: Structured Clinical Interview for DSM-IV Axis I Disorders
UBACC: University of California, San Diego Brief Assessment of Capacity to Consent Questionnaire
WHO: World Health Organization
